# Supersaturation-Based Drug Delivery Systems: Strategy for Bioavailability Enhancement of Poorly Water-Soluble Drugs

**DOI:** 10.3390/molecules27092969

**Published:** 2022-05-06

**Authors:** Arvind Sharma, Kanika Arora, Harapriya Mohapatra, Rakesh K. Sindhu, Madalin Bulzan, Simona Cavalu, Gulsheen Paneshar, Hosam O. Elansary, Ahmed M. El-Sabrout, Eman A. Mahmoud, Abdullah Alaklabi

**Affiliations:** 1 Chitkara College of Pharmacy, Chitkara University, Rajpura 140401, Punjab, India; arvind.sharma@chitkara.edu.in (A.S.); kanika20004.ccp@chitkara.edu.in (K.A.); priya20003.ccp@chitkara.edu.in (H.M.); gulsheen@chitkara.edu.in (G.P.); 2 Faculty of Medicine and Pharmacy, University of Oradea, P-ta 1 Decembrie 10, 410087 Oradea, Romania; bulzan.madalin@yahoo.com; 3 Department of Plant Production, College of Food and Agricultural Sciences, King Saud University, P.O. Box 2460, Riyadh 11451, Saudi Arabia; helansary@ksu.edu.sa; 4 Department of Applied Entomology and Zoology, Faculty of Agriculture (EL-Shatby), Alexandria University, Alexandria 21545, Egypt; elsabroutahmed@alexu.edu.eg; 5 Department of Food Industries, Faculty of Agriculture, Damietta University, Damietta 34517, Egypt; emanmail2005@yahoo.com; 6 Department of Biology, Faculty of Science, University of Bisha, P.O. Box 551, Bisha 61922, Saudi Arabia; alaklabia@gmail.com

**Keywords:** supersaturated drug delivery systems, lipid-based systems, solid dispersions, bioavailability

## Abstract

At present, the majority of APIs synthesized today remain challenging tasks for formulation development. Many technologies are being utilized or explored for enhancing solubility, such as chemical modification, novel drug delivery systems (microemulsions, nanoparticles, liposomes, etc.), salt formation, and many more. One promising avenue attaining attention presently is supersaturated drug delivery systems. When exposed to gastrointestinal fluids, drug concentration exceeds equilibrium solubility and a supersaturation state is maintained long enough to be absorbed, enhancing bioavailability. In this review, the latest developments in supersaturated drug delivery systems are addressed in depth.

## 1. Introduction

Currently, pharmaceutical pipelines are typically clogged with poorly water-soluble drug candidates, which encourages novel formulation technologies to offer dosage forms with appropriate biopharmaceutical characteristics. A novel drug delivery system approach using supersaturated drug delivery systems (SDDSs) to achieve adequate oral bioavailability is well documented and reported. Supersaturated drug delivery systems (SDDSs) incorporate the drug in a high-energy or quickly dissolving form, resulting in intraluminal concentrations exceeding the solubility of the drug in a saturated state. The formulated solution in a supersaturated state must be stabilized to allow for substantial absorption and eventually adequate bioavailability for the strategy to be effective. Precipitation inhibitors (PIs), which operate in several mechanistic ways, may be used to stabilize a supersaturated solution Ref. [1]. Supersaturated drug-delivery systems (SDDSs) such as amorphous solid dispersions (ASDs), nanoparticulate systems, and lipid-based delivery systems have been widely employed to improve the solubility and oral bioavailability of poorly water-soluble drugs (PWSDs). For a long enough time, supersaturation keeps the drug concentration in the gastrointestinal lumen above its equilibrium solubility, allowing absorption through the intestinal membranes. The “spring” and “parachute” effects underpin supersaturated systems. The spring effect is observed with thermodynamically unstable high-energy medicines, resulting in supersaturated drug solutions, whereas the parachute effect uses polymers or surfactants to sustain the supersaturated state for an acceptable time period [2].

Studies conducted for the last two decades on nano drug delivery systems have suffered demerits such as degradation of drugs at higher temperatures or the removal of solvent. It is evident from studies conducted by using supercritical carbon dioxide that problems associated with micro or nano drug delivery systems can be overcome. A pure substance is in the supercritical fluid state if its temperature and pressure conditions are above the critical point values. In comparison to other supercritical fluids, scCO_2_ has mild critical temperature (Tc) and pressure (Pc), i.e., 31.1 °C and 7.3 MPa, respectively. By varying process variables, supercritical carbon-dioxide-assisted technologies may produce a variety of morphologies and sizes, including nanoparticles and nanocrystals [3]. Several approaches for producing drug nanoparticles/nanocrystals based on the use of scCO_2_ as a solvent have been developed throughout the years. This category includes the following techniques: (a) rescue of supercritical solutions (RESS); (b) rescue of supercritical solutions with a solid co-solvent (RESS-SC). Until recently, scCO_2_-assisted methods have successfully decreased the microscopic sizes of a variety of active chemicals, both naturally occurring and chemically manufactured [4]. To regulate the release of the active component contained in the nanoparticles/nanocrystals, the APIs have been treated alone or with polymers/additives utilized as stabilizers, protective agents, and/or transporters [5]. In the presence of scCO_2_, polymers are also utilized to increase the solubility of pharmaceuticals in terms of recovery, shape, and/or particle size.

This review emphasizes the role of polymers in supersaturation maintenance in the solution state, the mechanistic approach of how polymers inhibit the nucleation and crystal growth, and enhancing the solubility and oral bioavailability of poorly water-soluble drugs through various supersaturated delivery systems.

## 2. Supersaturated Drug Delivery Systems

When completely exposed to the aqueous environment of the gastrointestinal system, supersaturable formulations will release a high enough concentration of drug to supersaturate the solution. For drugs to be absorbed in the timeframe required, supersaturation must be created and sustained. The ability of a formulation to form and maintain a supersaturated drug solution is determined by a variety of factors, including the manufacturing methods used, the physicochemical properties of the substance, and the propensity to form and maintain a supersaturated drug solution. The “spring and parachute” theory is a popular way of describing how to create and maintain a supersaturated state [6].

### 2.1. Precipitation Inhibitors

Optimizing a SEDDS to facilitate supersaturation of drug in colloids disseminated in the GI fluid can boost thermodynamic activity, resulting in a higher free drug concentration. As a result, this formulation strategy will improve drug absorption by promoting drug supersaturation and prolonging the supersaturation time in the GI fluid [1,7,8]. Pharmaceutical excipients that retard precipitation of drug, also known as PIs [9], are one method of preparing these formulations, as shown in Table 1.

**Figure 1:**
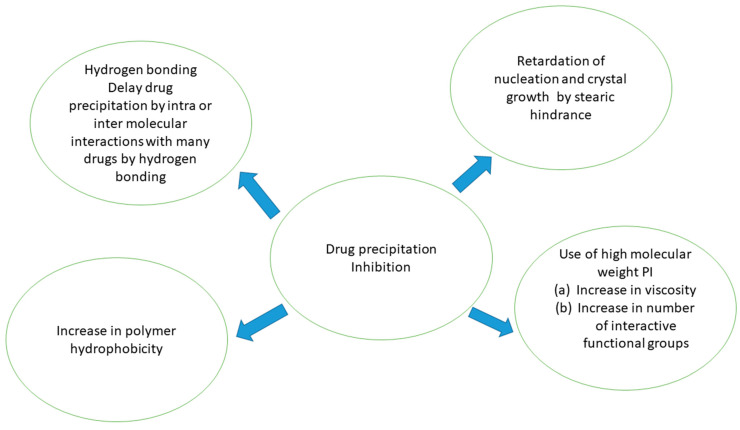
Mechanism of drug precipitation inhibition.

PIs have been shown to maintain a supersaturated metastable state for long enough to increase the absorption of PWSDs. Thermodynamic inhibition and kinetic inhibition are two mechanisms that can justify drug precipitation inhibition [10]. PIs usually work through a kinetic inhibition process. Drug precipitation (DP) may be kinetically inhibited by delaying drug precipitation from a drug solution in supersaturated state, as shown in Figure 1. Via interactions with drug molecules, crystal nucleation or/and growth can be inhibited by PIs. Furthermore, the action of PIs on the medium’s viscosity and pH can lead to drug precipitation inhibition [11,12]. Drug precipitation, on the other hand, can be thermodynamically blocked by increasing the solubility of drug. To enhance the drug solubility, numerous solubilizing agents reported in literature, such as CDs, co-solvents, and surfactants, can increase drug solubility, lowering the degree of supersaturation and thus the nucleation rate. PIs can also enhance solubilizing effects [13].

**Table 1:** Supersaturated drug delivery systems and precipitation inhibitors.

S.NO	Formulation (Spring Form)	Precipitation Inhibitors (Parachute)	Model Drug	In-Vivo and In-Vitro Performance (PK)	Reference
1.	Solid dispersion	PEG 6000, PVP, HPMC	Tacrolimus	HPMC When compared to crystalline powder administration, there was a 10 fold increase in C_max_ and AUC.	[14]
2.	self-emulsifying drug delivery systems (SEDDS)	PEG 6000, PVP, HPMC	Paciltaxel	HPMC resulted in a 20-fold increase in C_max_ and 10-fold increase in oral bioavailability	[15]
3.	solid dispersions	HPMC, HPMCAS L, M, H SOL	Candesartan Cilexetil	HPMCAS M presented good anti-precipitation efficacy in both media, reaching higher AUC maintainind drug supersaturation for up to 120 min	[16]
4.	Soild Dispersion	SOL, SLS, P188, PS20	Chlorthalidone	SOL-SLS complex impacted positively release and Physical stability of chlorthalidone	[17]
5.	Solid dispersion	SA, SLS		All SDs, demonstrated no drug recrystallization after 34 months of storage exception being those prepared with SA alone or SA-SLS at high drug loading	[18]
6.	Solid dispersion	P188, F127, SDS, HS15, ST and TPGS	Lacidipine	Nearly 3.3 and 3.7-fold increase in C_max_ and AUC (0–∞) respectively was attained with formulation based on LCDP/SOL/SDS	[19]
7.	Amorphous Solid dispersion	Eudragit EPO	Trimethoprin and sulfhmethooxazole	The 70% polymer formulation was able to produce and sustain the supersaturated phase of both compounds for 24 h. When compared to the combination of agents, improved antimicrobial effect was observed.	[20]
8.	Amorphous Solid dispersion	hydroxypropylmethylcellulose acetate succinate (HPMCAS) type M	Candesartan cilexetil	Reduced the desupersaturation of both drugs	[21]
9.	Amorphous Solid dispersion	Saccharin (SAC)	Griseofulvin	AUC increased 20% in comparison to conventional formulation	[22]
10.	Soild dispersion	HPMC	Magnolol	Increased the bioavailability (the relative bioavailability was 213.69%	[23]
11.	SEDDS	PEG 400, Tween 80, Miglyol 812 N	Carbamazepin	When compared to the commercial formulation, 200 mg of dosage resulted in 6.7 and 5.9 times larger increases in C_max_ AUC, respectively.	[24]
12.	SEDDS	HPMC-E5 PVP-12PF	Celecoxib	When comparison to solution and conventional capsule formulations, excellent IVIVC and Human PK was observed.	[25]
13.	SEDDS	Soluplus		In comparison to drug powder, there was a 2.34-fold increase in C_max_ and a 4.82-fold rise in AUC.	[26]
14.	SEDDS	Soluplus, PVP VA64, poloxamer 407, PEG 6000	Celecoxib	PI effect of Soluplus is greater than PEG 6000 PVP, VA64, poloxamer 407 & PEG 6000	[27]
15.	SEDDS	Eudragit E PO	Curcumin	A 50 mg/kg dose of PI resulted in a 1.22 and 53.14-fold enhancement in absorption in rabbits when compared to the aqueous phase and standard SEDDS without PI, respectively.	[28]
16.	SEDDS	Polyvinylpyrrolidone (PVP), hydroxypropyl methyl cellulose (HPMC)	Curcumin	The increased concentration-dependent effect was observed for PVP-K30 when used as PI in comparison to PVP-K90 without PI &HPMC.	[29]
17.	SEDDS	HPMC K100	Docetaxel	When SD rats were given a dose of 10 mg/kg, their AUC jumped by around 8.77 times which was 1.45-fold higher than the increases seen with the powder medication and traditional SEDDS without PI.	[30]
18.	SEDDS	HPMC (5%, *w*/*w*)	Ginger extract	SD rats were given a dosage of 100 mg/kg in experimental model of animals, 6-gingerol and 8-gingerol had three time the antioxidant activity (BA) of the unformulated extract, i.e., control rats.	[31]
19.	SEDDS	HPMC-E5 (5%, *w*/*w*)	Glipizide	AUC (2.7-fold) and C_max_ (3.4-fold) were found to be increased in Himalayan rabbits when solid su-SEDDS were administered at a dose of 1 mg/kg as compared to the standard drug.	[32]
20.	SEDDS	Poloxamer, HPMC	Griseofulvin	Aqueous suspension showed three-fold less permeability through the intestinal tract of Wister rats when given a dose of 1 mL at a concentration of 0.05 mg/mL (0.05 mg/mL).	[33]
21.	SEEDS	HPMC, PEG 4000, PVP-K17	Indirubin	When compared to the SEDDS without PI, the chemical exhibited better oral absorption and relative BA [129.5%] when delivered in vivo to SD rats at such a dose frequency of 2.58 mg/kg.	[34]
22.	SEDDS	HPMC-E5LV	Paclitaxel	Compared to the Taxol^®^ formulation and the standard SEDDS, the SD rats administered optimised formulation. At a dosage of 10 mg/kg, the C_max_ and AUC were ten-fold and twenty-fold higher, respectively.	[35]
23.	SEDDS	HPMC-E15LV	Resveratrol	After 20 mg/kg administration to Wistar rats, the su-SEDDS demonstrated a 1.33-fold increase in AUC compared to standard SEDDS lacking PI.	[36]
24.	SEDDS	HPMC-E50LV	Silybin	SD rats were given a dosage of 533 mg/kg, which resulted in a 3-fold increase in AUC compared to the usual SEDDS without HPMC in vivo.	[37]
25.	SEDDS	Poloxamer 407, Poloxamer 407 > HPCD, Eudragit L100 HPMCP	Silymarin	Using a dosage of 28 mg/kg of silybinvsLegalon^®^ (a commercialized product) and a 76% BA of su-SEDDS concentration, silybin was evaluated in vivo in rabbits.	[38]
26.	SEDDS	Soluplus, HPMC, PVP	Tacrolimus	As with conventional SEDDS, the Area under curve and C_max_ of su-SEDDS at 1 dose of 5 mg/kg in SD rats were equivalent or larger than conventional SEDDS at the same dosage.	[39]
27.	SEDDS	Poloxamer 407	Valsartan	Using a dosage of 10 mg/kg, the medication was put to the test in SD rats. AUC ranges between about 177 and 198%when compared to API and Diovan^®^, a commercial product.	[40]
28.	SNEEDS	HPMC, PVP, PVP/VA, and Soluplus^®^	Aprepitant	Increased dissolution rate of the drug due to enhanced solubility	[41]
29.	SEDDS	HPMC E5	Quercetin	improved AUC and C_max_ values in comparison to conventional SEDDS	[42]
30.	SNEDDS	HPMC	Albendazolum	Enhancement in the solubility and oral bioavailability	[43]
31.	SNEDS	Poloxamer 407 (P 407), Eudragit^®^ L100-55 (Eu), Kolliphor^®^ HS15 (KHS15), Kolliphor^®^ RH40 (K RH40), vitamin E TPGS (vit E TPGS) & Soluplus^®^	Cinnarizin	2.7-fold increase in AUC 0–24 h	[44]
32.	SEDDS	Cremophor RH40 & Macrogol 200	Cepharanthine	Relative bioavailability was 203.46%	[45]
33.	SNEDS	Polyoxyethylene (80) sorbitan monooleate (Tween^®^ 80), d-α-tocopherol polyethylene glycol 1000 succinate (d-TPGS, Tocophersolan)	Celecoxib & fenofibrate	SNEDDS development in a short time with manageable resources	[46]
34.	SMEDDS	PVP	Biphenyl dimethyl dicarboxylate	Significantly increased the C_max_ and AUC	[47]
35.		HPM C, HPMCA, SPV Pluronic F108,	Venetoclax	In vivo exposure of venetoclax was achieved	[48]

HPMC: Hydroxypropylmethylcellulose; SLS (sodium lauryl sulfate); (Kolliphor P188^®^); Kolliphor PS20^®^; vitamin E polyethylene glycol succinate (TPGS); Solutol^®^ HS15; Kolliphor^®^ P407 (F127); (HPMC) hydroxypropylmethylcelluloseacetate succinate (HPMCAS); H, M, and L-sodium dodecyl sulfate (SDS); SA: sodium alginate; SOL: polyvinyl caprolactam-polyvinyl acetate-polyethylene glycol graft copolymer.

Precipitation inhibitors are broadly classified in two main categories, polymeric PIs and non-polymeric PIs. Essentially, most PIs are polymers. As a result, they may be classified as polymeric PIs (PPIs) [11]. PPIs are broadly classified as (1) surface-active and (2) non-surface active. Cremophor EL, polyethylene glycol 1000 succinate (TPGS), D-tocopherol and poloxamers are examples of surface-active PPIs. Concentrations exceeding critical micelle concentration (CMC) are primarily responsible for surface-active PPIs’ precipitation inhibition effect, and further, they may improve the drugs’ solubility in equilibrium state. Surface-active PPIs can improve the bioavailability of PWSDs by maintaining supersaturation and providing high equilibrium solubility. If surfactants are adsorbed on a surface of a nucleus, however, there may be an undesired increase in nucleation rate. The interfacial tension between the surface and solvents is reduced if surfactant is present. PPIs (non-surface active) are further categorized: cellulosic and non-cellulosic [42,43]. CMC, MC, HPMC-AS, acetate phthalate, alginic acid, cellulose, HPC, hydroxyl ethyl cellulose, Na-CMC, HPMC, and arabic gum are cellulosic PPIs; poly(vinylpolypyrrolidone), polyvinylpyrrolidone vinyl acetate, polyvinyl alcohol and Eudragit are non-cellulosic PPIs. Using PVP and its derivatives to avoid precipitation of pharmaceuticals manufactured as supersaturated solid particles and SDDSs has been shown to be an effective method.

Crystal growth and nucleation rates were shown to be slowed by CDs [11]. In addition, recent investigations have shown that CDs can stabilize supersaturated medication solutions, avoiding precipitation of the drugs [45,47]. CD inclusion complexes with various PWSDs can enhance solubility of drugs while lowering the ratios of supersaturation. In the presence of CDs, the drug solution supersaturated state could be maintained for at minimum 2 h. CDs, according to the authors, can prevent drug precipitation both thermodynamically and kinetically by increasing saturation solubility (apparent) and interacting via hydrogen bonding with API. Furthermore, it is hypothesized that CD’s can result in the stabilization of solutions in a supersaturated state regardless of drug precipitation by increasing viscosity and diffusion resistance and improving the cohesive nature of water [48]. Various supersaturable formulations have shown similar solubility improvements and precipitation inhibition actions via CD supersaturation stabilization [49,50].

#### Precipitation Inhibitors’ Influence on Supersaturation

The supersaturation is maintained using a variety of polymers. Precipitation inhibition depends on a combination of drug and polymer properties. Due to the presence of hydroxyl (OH) groups that may interact with drug substances, cellulosic polymers are most commonly used [51]. Curatolo et al. evaluated nine drugs against 41 polymers and observed that HPMCAS was perhaps the most promising at maintaining supersaturation [52]. Bile salts, in addition to cellulose-based polymers, may be used as PIs [53]. Chen et al. looked into the efficiency of bile salts in maintaining supersaturation on 11 different drugs. Inhibition of nucleation was observed for all drugs, indicating that they could be explored as PIs for supersaturated formulations [54]. For supersaturation maintenance, some studies show that using combinations of polymers instead of single polymers is beneficial. Prasad et al. evaluated the combined effect of Eudragit E100 and PVP K90 on the supersaturation retention of indomethacin. According to 1H nuclear magnetic resonance (NMR) studies, Eudragit E 100 inhibited nucleation by interacting with indomethacin, whereas PVP K90 inhibited crystal growth by adsorbing onto the crystal surface [55]. The combined effect of polymer and surfactant on the precipitation inhibition of ASD chlorthalidone was investigated by Franca et al. The polyvinyl caprolactam–polyvinyl acetate–polyethylene glycol grafted co-polymer and sodium lauryl sulphate were used to stabilize chlorthalidone, which belongs to BCS class IV. Polymer, surfactant, and a combination of the two were used to create solid dispersions. Solid dispersions were made using polymer, surfactant, and a combination of the two. Water and biorelevant media were used to characterize the solid dispersion regarding in vitro dissolution in sink and non-sink conditions. Solid dispersion stability was tested under various storage conditions. Under non-sink conditions, the ternary system of chlorthalidone shows fast release of >80% in 15 min and maintains supersaturation for 6 h. At various storage conditions, the system was found to be stable [56,57]. Beverage et al. investigated how polymers (HPMC-P, HPMC-AS, HPMC-E5, HPMC-E4M, HPMC-P, and HPMC-E50) affected the supersaturation of drugs such as etravirine, danazol, loviride, ritonavir, and fenofibrate in simple buffer, aspirated human intestinal fluid, and relevant media. To induce supersaturation, the solvent shift method was used, and cellulosic polymers showed more effective inhibition. The polymer was ineffective in the simple buffer, and biorelevant media confirmed its ineffectiveness in human intestinal fluid, indicating that simulated fluid can be used to establish a correlation with in vivo conditions. Human gastric fluid, simulated gastric fluid, and fasted state simulated gastric fluid were used as precipitation media in a study by Beverage et al. [58]. Model drugs included loviride, glibenclamide, itraconazole, danazol, and etaravine; and PIs included Eudragit^®^ E PO, HPMC-E5, and PVP K25 [59]. The solvent shift method was used to create supersaturation; Eudragit E PO and HPMC-E5 had a moderate effect, but PVP K25 had no effect. According to the findings, supersaturation in gastric fluid is limited, and it is preferable to investigate supersaturation in intestinal fluid [60]. Using atomic force microscopy [61], Schramet et al. investigated the effect of polymer conformation as a function of pH on felodipine precipitation inhibition [62]. At two different pH levels, HPMCAS was used as a PI (3 and 6.8). At the lower pH, HPMCAS was found to be less effective, whereas at the higher pH, it was found to have more inhibitory activity. Polymers in their ionized forms become self-repulsive, causing the chains to extend, whereas polymers in their unionized forms coil due to intramolecular hydrogen bonding between polymer molecules. In this case, pH influences polymer conformation and thus its inhibitory potential [63,64].

### 2.2. Supersaturated Drug Delivery Systems (SDDSs) (Spring Form)

A drug in a higher energy state (“spring form”) is normally used to develop a thermodynamically unstable, supersaturated drug solution. Formulations that can enable the formation of a supersaturated state are usually classified into two groups: high-concentration drug solutions and high-energy or fast dissolving solid forms are achieved by modifications in wettability, morphology, or particle size [65] (Table 1). These formulations include amorphous morphologies, co-crystals, and salt crystal forms of crystallized crystals; co-solvent nanoparticles, systems, and solid solutions; or dispersed formulations with nanoparticles. These methods have been thoroughly examined in the literature [1,66]. Methods such as solid solution dispersion and lipid formulation have gained a great deal of interest.

#### 2.2.1. Solid Dispersion-Based Supersaturated Drug Delivery Systems (SDDSs)

**Figure 2:**
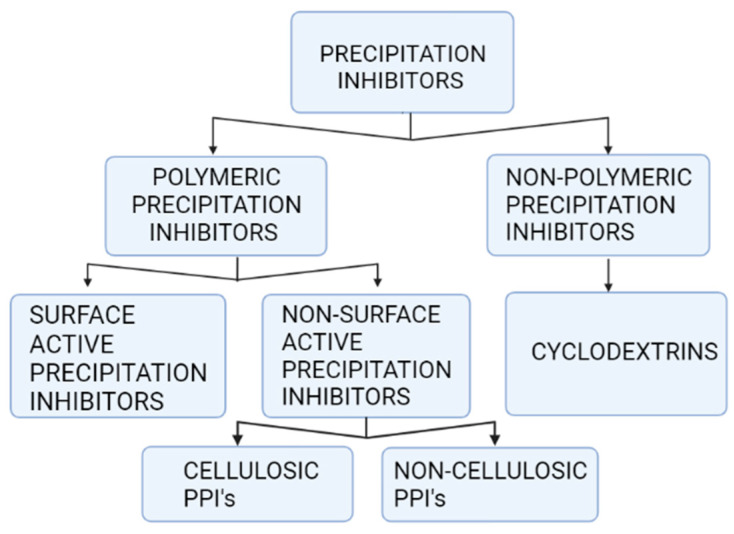
Precipitation inhibitors.

Despite the fact that poorly soluble drug candidates (nearly 40%) account for the majority of APIs under research and development, they have the highest drop-out rate [67] owing to low oral bioavailability [68]. Drug delivery in amorphous solid dispersion (ASD) drug delivery systems is one possible solution [69]. ASDs have piqued researchers’ interest in recent decades, as evidenced by patent analysis and a recent literature survey. An exponential increase in articles and patents has been observed in both academia and industry [70]. ASDs are thoroughly reported to improve both in vitro performance and in vivo efficacy in animals [71,72,73,74,75,76,77,78,79,80] and in humans, when used in oral drug delivery. Recent statistical meta-analyses [81,82,83,84,85,86,87,88,89,90,91,92] found that ASD has a statistically positive effect on bioavailability. Only 24 ASD formulations were available on the market in 2015 in comparison to 3732 registered drug products (2019). These account for approximately 0.6% of drugs on the market. Bioavailability in animals decreased or was unchanged in 18% of ASD formulations [93,94]. This may be because ASDs are more complex systems than standard medication formulations. ASD formation with a particular polymer is not assured using an API because mixing or dissolution can be time-consuming and complicated. After the product formation, shelf life is still an essential consideration, since crystallization can occur after manufacture [95]. It is critical to have predictive techniques and models to minimize the attrition rate of PWSDs. Such methodologies and insights allow for feasibility estimations without or with only a few trials. Such approaches are pretty limited in their applicability to ASDs. The ability of an API to be administered as an ASD is more critical in determining whether or not to pursue the development of poorly soluble therapeutic drug candidates. When considering the possibility for enhanced bioavailability of a drug in the future, it is critical to estimate its potential. It is crucial to reduce development costs [92,93,94,95,96,97]. When an ASD contacts the aqueous media, it spontaneously dissolves into a conventional solution (molecularly dissolved API). There are several steps involved in the process of API release from solid ASDs, which will be explored in further detail in the parts of this review that follow. We use the collective term “colloidal system” to describe all the states that form due to the ASD in dissolved form. The following is a description of the processes of dissolution and uptake: The intake of drugs from the ASD and API dispersion and the stabilization of ASDs are all under investigation. Several different terms are used in the contemporary literature to refer to the solubilization and supersaturation of APIs in solutions. According to a general definition, the solubility of API molecules that are distributed molecularly in an aquatic solution is known as “solubility.” Solubility is defined as the quantity of API that can become molecularly dispersed in an aqueous solution of a certain volume. We propose to use terminology based on the work of Taylor and Zhang [98]. The maximum amount of API that is detectable in solution, we use in this article to refer to the amorphous–aqueous phase separation (AAPS). We use AAPS as a combination of those two phenomena, which many authors do not distinguish between. As it is a thermodynamically metastable form, the drug will crystallize at some point during this AAPS stage. We use the term AAPS for particles resulting from AAPS. A supersaturated solution is a solution wherein crystallized particles appear because solubility has been exceeded by APION dissolution. The amount of API that can be detected in the solution can be measured in API molecules. This article uses the terminology shown in Figure 2. Based on the research literature, Solubility is typically defined in terms of apparent solubility, which relies heavily on the method employed to estimate it. In order to improve the release mechanism from solid ASDs, physicochemical factors must be identified. When dissolving ASDs, the formation of colloids might be difficult to characterize [99,100,101].

From a physicochemical perspective, AAPS occurs only when the supersaturated concentration of drug exceeds solubility in the amorphous state [98,102,103]. However, polymers play a significant role in the establishment of AAPS particles and separation from pure solvent API mixtures. For example, from NMR observations of nifedipine and HPMC derivatives (HPMC acetates and succinates), it was interpreted that more the hydrophobic the polymer (relying on pH and the polymer derivatives), the greater the distribution of polymer into the AAPS. Polymers were able to minimize crystallization in these particles [104].

An investigation of a HPMC–AS (Posaconazole) system generated results similar to those obtained in the previous study [105]. Using Poloxamer 407 and PEG 6000, researchers demonstrated significantly improved in vitro bicalutamide performance [106]. When crystallinity in a polymer matrix was reduced, either completely or partially, it had no impact on the effectiveness of ASDs. The authors concluded that improved solubilization and wetting of the API-contained inside nanoaggregates were the underlying causes of the improved dissolving capabilities. A variety of complex mechanisms control the crystallization of drugs from supersaturated solutions and their kinetics [102].

Furthermore, when polymers are present in solution and can further stabilize the system, it is crucial to maintain the drug in the supersaturated state in solution [107]. One group studied the impact of polymers on hardening from supersaturated fluids using wide-angle X-ray scattering and synchrotron radiation. The pure drug’s crystallization times were demonstrated. Six dihydropyridine calcium channel blocker solutions were extremely heterogeneous. That study examined the behavior of 51 different medications in dissolved, amorphous states, and found the results to be in agreement. The time it takes for a drug to crystallize varies significantly between drugs, indicating that a higher molecular weight leads to faster crystallization [108,109,110,111].

Curatolo et al. investigated 41 possible PIs, some surface-active and others not, for stabilizing 9 distinct APIs in a supersaturated state [47]. Some of the fundamental concepts of polymer-stabilizing supersaturation in ASD have been investigated independently for last the decade. These principles include changes in solubility and viscosity (solution properties), and stabilization requires changes to the crystal’s adsorption layer (i.e., diffusion through the layer). Curatolo et al. discovered several mechanisms that cause polymers to inhibit crystallization. They described the precipitation inhibition effect based on physicochemical interactions [112,113]. ASDs formulated using mefenamic acid in Eudragit EPO were found to result in increased solubility [114]. NSAIDs such as piroxicam and indomethacin also improved solution stabilization. Different molecular interactions (hydrophobic, hydrogen, and ionic bonds) between drug and polymer were observed. These interactions are most likely to facilitate supersaturation [102].

Mosquera-Giraldo et al. (2018) [115] investigated the effects of nine compounds (nevirapine, atazanavir, nifedipine, celecoxib, and ritonavir) in five different synthesized cellulose derivatives in the presence of five distinct synthetic cellulose derivatives. Polymers having a short side chain and only single carboxylic terminal group are the most effective at preventing drug crystallization. A less successful class of polymers is those with longer side chains and two carboxylic terminal groups. For a polymer to be more efficient, higher chances of interaction among drug and polymer must exist.

There is a more significant possibility of drug–polymer interaction with more effective polymers. Delaying crystallization is most effective with polymers with intermediate hydrophilicity. Polymers having good aqueous solubility or PWS do not have the same outcomes in term of efficacy. Novel cellulose derivatives are the best at expressing this effect. On ritonavir crystals, these properties are critical for polymer absorbance. One study [51] reported the effect (41 PIs tested on 9 drugs). The study looked at the ability of 34 polymers to inhibit crystallization based on their chemical and physical characteristics. The authors concluded that contact between the polymer and hydrophobic drug-rich phase is essential for supersaturation stabilization.

A high-energy (amorphous) API is either dissolved or dispersed in a matrix made up of a soluble polymer or a combination of polymer and surfactant in the ASD API. The ASD produces supersaturated concentrations intraluminally of poorly aqueous-soluble drugs through many processes/mechanisms. Incredibly quick supersaturation is achieved via improved molecular solubility, higher apparent solubility owing to micellar solubilization, an increased rate of absorption, and increased SA being accessible for the absorption of distributed amorphous drug particles, to name a few reasons. According to new mechanism-based studies by Warren et al. [116,117,118,119], an aqueous medium containing melt extrudate [100] as a dispersion effects the instant generation of nanoparticles or microparticles in amorphous form, which are not required for long-term super saturation [117]. Other researchers have used the terminology LLPS, i.e., liquid–liquid phase separation, to describe a similar mechanism, but they consider the separated nano/microparticulate phase a constraint rather than a driving force for enhanced permeation [118]. Micellar solubilization (enhanced apparent solubility), on the other hand, was observed not to enhance permeation. The popular meta-analysis conclusions show that ASD is an encouraging way to improve oral bioavailability consistently. This method is rated three in terms of absorption rate and tmax values (more moderate than lipid-supersaturable lipid-based formulations). It achieves fast absorption with a tmax ratio of 0.66 (95% CI, 0.49% and 0.84). Furthermore, this SDDS formulation theory has been the most thoroughly researched in recent years: a total of 83 publications between 2010 and 2015, accounting for nearly half of all recorded SDDS formulation works. The popularity of amorphous solid dispersion, along with its promising properties, allows this SDDS formulation theory to be a viable solution to the problem of low water solubility.

#### 2.2.2. Supersaturable Lipid-Based Formulations

Supersaturable lipid-based formulations are beneficial for absorption of drugs that are water-insoluble or poorly water-soluble, as they encourage solubilization and supersaturation [119]. Drug systems that self-emulsify and dissolve in water are well-known lipid-based formulations, consisting mostly of cosolvent, surfactants, and oils. Some researchers have tried to expand the supersaturated phase in the small intestine after intestinal fluids were dilute using a self-emulsifying formulation, but this has not been successful yet. S-SEDDSs is a term used to describe these formulations, which contain PIs such as polymers to retain the supersaturated phase. A fundamental strategy to enhancing the inhibition of precipitation is the development of supersaturating microemulsifying drug systems (S-SMEDDSs) and self-nanoemulsifying drug systems (S-NEDDSs), which use microemulsions (droplet dimensions 100–250 nm) and nanoemulsions (droplet dimensions 100 nm), respectively. Beyond solubilization and supersaturation, the enhancement of the oral bioavailability given by LBDDS is determined by the lipid digesting process, which includes contact with bile and the possibility of absorption through the lymphatic pathway. These characteristics distinguish this formulation strategy from other SDDS formulations. In comparison to the other SDDS formulations, the supersaturation and oral bioavailability improvements obtained by supersaturate lipid-based formulations were the most moderate, and in several cases, below average. The fact that the degree of oral bioavailability improvement was not extremely ideal does not negate the fact that this formulation idea provides one significant advantage: the amount of variability between studies was observed to be minimal. The accuracy of formulation output during preclinical development is critical. This method is highly beneficial due to the high uniformity in oral bioavailability improvement by supersaturable lipid-based formulations in a supersaturated state. Some classes of Eudragit, PVP and Poloxamer, can be dispersed in the lipid phase, according to Ilevbare et al. [120,121,122]. Jang et al. developed su-SEDDS carbamazepine formulations [120]. They demonstrated that adding 2% PVP as PI to the su-SEDDS formulation effectively maintained the supersaturated state and slowed precipitation kinetics. The average particle size of the developed emulsion following su-SEDDS dispersion was approximately 34 nm, and the su-SEDDS in vitro drug release rate was much higher than the rate of a commercial tablet, indicating that the su-SEDDS dispersion was effective. In terms of pharmacokinetic properties, the su-SEDDS had a C_max_ that was 6.7 times greater and an AUC that was 6 times higher than those of the commercially available tablet.

In the case of silymarin, Poloxamer 407 was confirmed to be the most effective PI for the production of a stable su-SEDDS formulation. When compared to a commercial product, Legal on, the su-SEDDS had a relative bioavailability of roughly 760%, indicating that it was highly bioavailable. In the study presented in [123], because most PIs are water-soluble, SEDDSs (supersaturated state) were investigated which had PI dispersed in the oil phase. The first SEDDSs investigation was reported for paclitaxel [124], a taxol derivative for treatment of cancer, using HPMC as the PI [125].

In vitro, it was reported that a formulation containing 5% (*w*/*w*) HPMC extended the supersaturated state for two hours, related to quick drug precipitation in typical SEDDS without HPMC [126]. When compared to the formulation without PI, this paclitaxel [123] SEDDS resulted a ten-fold enhancement in oral BA approximately (10%) in rats in a PK trial. A recent investigation confirmed that the above-discussed PI is effective for AMG 517 SEDDSs. A non-surface-active PI, i.e., PVP (K30), on the other hand, had no effect on precipitation. SEDDSs with HPMC as a PI have also shown comparable improvements in oral BA for curcumin, silybin, and PNU-91325. When raloxifene hydrochloride is combined with L-HPC, it has been observed to have a greater solubility and dissolving rate [127].

**Figure 3:**
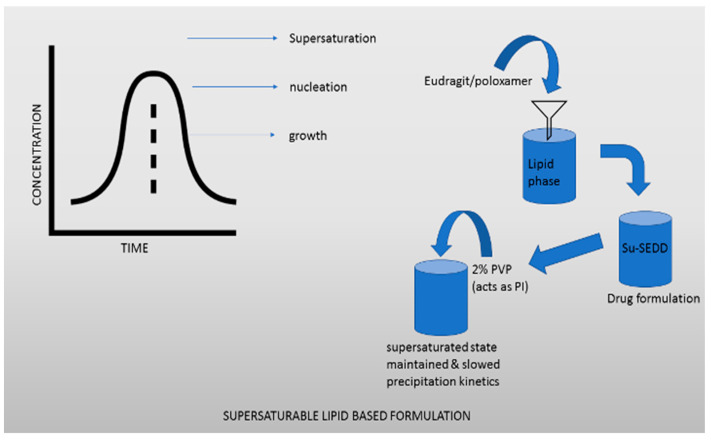
Supersaturable liquid base formulations.

An oil–water microemulsion was formed after a pH 2.5 formulation was compared to a typical tablet. This resulted in an increase in the release of the medication when compared to the standard tablet formulation. The SEDDS of celecoxib [128] used Soluplus^®^ as a PI. There was evidence this SEDDS allowed the fastest dissolution (about 90%) and delayed drug crystallization. The SEDDS had the highest C_max_ and the shortest Tmax, indicating that it had the fastest and most improved absorption. Traditional SEDDS and Su-SEDDS had relative BAs of 263% and 355%, respectively, as compared to celecoxib suspension [129,130]. According to Jaisamut et al., the pharmaceutical ingredients in their optimal curcumin SEDDS were 40% oils, 55% surfactants, and 5% Eudragit^®^ E PO. Compared to the normal SEDDS and aqueous suspension groups, the su-SEDDS-administered group demonstrated 1.22- and 53.14-fold greater curcumin absorption in rat PK studies. SEDDS significantly increased curcumin’s permeability in a Caco-2 monolayer when compared to an unformulated curcumin suspension [128], prolonging precipitation and keeping indole concentrations high in water after diluting Su-SEDDS with 0.5% PVP K17, according to Chen. SEDDS PK experiments in male rats found that bioavailability was 1.4 times better with a PI than with traditional SEDDS [131,132]. To increase the oral bioavailability of dutasteride, a water-soluble medication, Baek et al. developed a gelatin microparticle containing SEDDS using a spray-drying technique. Researchers looked at a variety of variables, including the amount of gelatin and hydrophilic additions. In vitro precipitation dissolution studies suggested that gelatin might be used as a solidification active ingredient and a pharmaceutical ingredient to generate dutasterside su-SEDDSs [133]. A combination of SEDDS with gelatin microparticles and Soluplus^®^ also resulted in better dutasteride dissolution and oral absorption when compared to the individual parts [134]. Using solvent evaporation and Santa Barbara Amorphous-15 (SBA-15) as the solid carrier, an optimized solid SEDDS accommodating fenofibrate was constructed. Pharmacokinetic studies in beagle dogs showed that the AUC of SEDDS with Soluplus^®^ was 1.4 times greater than that without Soluplus^®^ [135]. Figure 3 illustrates the benefiting drugs and precipitated inhibition effects of cyclodextrins as both springs and parachutes in the systems.

Water-soluble drug carriers can also be chemically inert particles. In an effort to improve the dissolving process, silica materials, notably structured mesoporous silica, have been studied. Ordered mesoporous silica has cylindrical pores (diameters ranging from 2 to 50 nm) that look parallel to each other and are separated by thin walls of silica. The intraluminal concentration being supersaturated occurs from competing adsorption and inflow of solvent (water) onto the materials’ surfaces. This formulation principle has two distinct advantages over other SDDS formulations: (1) it is capable of physically stabilizing amorphous and drugs dispersed molecularly; (2) it can manipulate the pore size of silica materials to control the supersaturation rate. The tmax proportion of SBS (silica-based systems) against crystalline pharmaceuticals was “0.80 [95% CI 0.88, 0.74], which is regarded as close to 1 according to meta-analysis data. SBS provides advantages for controlled medication release rather than fast absorption [134].

However, in vitro supersaturation was observed to be very variable, as indicated by the large interquartile assortment from the box plot. Silicate-nano matrix system research was the source of an incredibly high SRM (outlier). This work used a colloidal silica nano-matrix covered with a polymeric solubilizer to increase the water solubility of the medicinal ingredient dutasteride, yielding a supersaturation proportion of 326-fold that could be sustained for 12 h. Only one of the SBS formulations (SDDS) attained a substantial fraction of SRM values that ranged from 2 to 10.5, which are much lower than those for the other formulations. It was shown that the silica-based system (which comprises mesoporous silica, lipid hybrid systems, and nano matrix systems) ranked third in terms of in vivo oral bioavailability among supersaturated DDS formulation principles evaluated. As a “new” technology, SBS is projected to see a great deal of study in the next decades, which might provide more convincing proof of its efficacy in improving bioavailability [136].

#### 2.2.3. Transdermal Drug Delivery Systems (TDDSs)

For last three decades, supersaturating in TDDSs has been investigated and proven to be efficient [137,138,139]. However, supersaturation requires the selection of appropriate solvents or co-solvents [140]. Ibuprofen crystallization was prevented for more than a year when methacrylic acid composites (Eudragit^®^ RL, EuRL, EuE) were used as ibuprofen PI blockers in the matrices [141]. Another study used 25% polyethylene glycol (PG), 5% vitamin ETPGS, and 5% ethylene oxide/propylene oxide chunk copolymer (poloxomer188) solvents, and their combinations, to achieve various DSs (0.5, 1, 2.5, 5.0, 10.0, 25.0, and 50) [142]. Polymeric stabilizers such as HPMC and PVP K-30 were used in the vitamin ETPGS/ibuprofen combination to avoid crystal formation (HPMC showed better crystal growth inhibition than PVP K-30). PVP K-30 improved a medication’s skin penetration when compared to HPMC. A synergistic effect of flurbiprofen permeation across human skin and the chemical enhancer oleic acid was identified. An enhancement ratio (ER) of 9.9 was reported when both oleic acid and supersaturation were used together. When compared to EtOH, the 6-fold super saturated solutions had an ER of 4.5 [143]. Caffeine and sumatriptan transdermal films have also been found to create a supersaturated drug state on the skin surface, leading to enhanced penetration. Due to the development of HC in the skin, the drug’s thermodynamic activity increases [144,145]. The transdermal flow of a non-entrapped hydrocortisone (HC) liposomal supersaturated system has been demonstrated to increase, resulting in a supersaturated state [146].

## 3. Solid Dispersion-Based Supersaturated Drug Delivery Systems (SDDSs) vs. Supersaturable Lipid-Based Formulations

The medication is disseminated or dissolved in an amorphous matrix made up of (soluble) polymer or a polymer–surfactant mixture in amorphous solid dispersions. Through a variety of methods, amorphous solid dispersions produce supersaturated intra-luminal concentrations of poorly water-soluble medicines. Spontaneous supersaturation due to increased molecular solubility, a micellar-solubilization-induced increase in apparent solubility, a faster dissolution rate, a carrier-induced increase in wettability, and an increased surface area available for the dissolution of dispersed amorphous drug particles are just a few of them. According to Frank et al. latest’s mechanistic research, dispersion of a molten extrudate in an aqueous media result in the spontaneous creation of amorphous nano/microparticles, which are required for long-term supersaturation [117]. Others use the terminology liquid–liquid phase segregation to describe the same issue, but they regard the segregated nano/microparticulate phase as a barrier rather than a driving factor for improved permeation [118]. Supersaturated medication delivery systems are now in use and have a bright future ahead of them.

## 4. Conclusions

ASD-based drug delivery systems and super saturable lipid-based formulations are emerging techniques for improving the bioavailability of PWSD that induce and stabilize a drug in a supersaturated state in the GI fluid using PIs. This technique circumvents the major drawbacks of conventionally solubilized drug delivery systems.

Despite advancements in drug delivery systems, there are still undiscovered areas that need to be researched further in order to establish this method as a complete technology that can be used to build a successful end product. To employ these techniques for a target medication, one must first grasp the intricacies of the precipitation mechanism caused by the drug’s supersaturation. Based on this technique, it may be able to limit precipitation and sustain supersaturation by considering the numerous parameters that govern precipitation. As a result, those elements were included in our analysis.

The effects of changes in the GIT’s physiological state, caused by a variety of variables, on the performances of numerous super saturable ASD, SDDS, SBS, and TDDS formulations are well documented. However, the unwanted effects associated with PI interactions with lipid-digestion products, lipases, and lipids on drug candidates’ super saturation and absorption in the GIT were not addressed in earlier investigations but were examined in present review. For the production of desirable drug delivery systems, several additional aspects must also be taken into account [147,148,149].

Extensive experimental research into the performances of polymers during this process is still desired, and the process is currently poorly understood. Furthermore, it is uncertain whether PIs must be totally dissolved or kept in a colloidal condition to act effectively. This review also included many methods for adding PIs, and examples of medicinal application situations for each strategy. However, the mechanism of dispersing PI in lipids (high or low energy) and the effect of particle size on outcome, particularly in vivo performance, are yet unknown. Another risk element to consider is if the suspended PI powder acts as a seed to accelerate the nucleation process. Furthermore, its non-miscibility in liquid SEDDSs could pose problems about phase separation during su-SEDDS storage [150,151,152,153]. This effect may have occurred in prior su-SEDDS research involving hydrophilic polymers such as HPMC or PVP.

## Data Availability

The data supporting the findings of this study are available within the article.
